# Physical Culture

**Published:** 1890-09

**Authors:** 


### PHYSICAL CULTURE.

The following, from a professor in a well known university, shows to what extent physical development conduces to right living, and a recognition, by practical demonstration, that all infraction of these laws are paid for in physical deterioration:

"At present I do not know in my acquaintance with the students, which extends perhaps to half the members of the University, a single case in which the young man can be called a drunkard. I believe this gain to be due in large measure to the sense of pride in a physical state which affects by far the larger part of the students. Their experience in training, which is undergone in one way or another by a very large part of the young men, gives them by experiment a clear understanding as to the influence of hygienic conditions.

"In a similar way the use of tobacco has diminished. Between 1865 and 1880, it was not uncommon to find men so sodden with tobacco that they were unpleasant subjects to have in a small lecture-room. In this decade I have found but two or three persons affected to this extent by tobacco. Even the use of tea and coffee, on the whole undesirable with youth, but extremely common in former years, has remarkably diminished. I am informed that only about one-half the students indulge in these beverages. In fact, the ways of the trained men in a college, like the customs of any army in a State where the military arm has great importance, are effective upon the body of the folk. Reasonable living is necessary to athletic success, and the habits of these men become in a way a pattern for the school life."

